# The WHO's new End TB Strategy in the post-2015 era of the Sustainable Development Goals

**DOI:** 10.1093/trstmh/trv108

**Published:** 2016-02-16

**Authors:** Knut Lönnroth, Mario Raviglione

**Affiliations:** Global TB Programme, World Health Organization, Geneva, Switzerland

**Keywords:** Determinants, End TB Strategy, Social determinants, Social protection, Sustainable development goals, Tuberculosis

## Abstract

The WHO's new End TB Strategy 2016–2035 has evolved from previous global strategies to respond to old and new challenges and take advantage of new opportunities. It frames the global fight against TB as a development, social justice and human rights issue, while re-emphasizing the public health and clinical fundaments of TB care and prevention. In this commentary, we outline how TB prevention, care and control will both benefit from and contribute to the achievement of the new Sustainable Development Goals that were recently adopted at the United Nations.

Global development has entered a new era. World leaders are committing to ‘end poverty, promote prosperity and people's well-being while protecting the environment by 2030’.^1^ Seventeen new Sustainable Development Goals (SDGs) were adopted at the United Nations Summit in September 2015. The SDG era began on 1 January 2016; at the same time that the WHO's new End TB Strategy to prevent, control and end the TB epidemic came into action.^2^

The SDGs build on the unfinished Millennium Development Goals (MDGs) agenda, but with a broader scope and more ambitious targets. They cover three dimensions: economic, social and environmental. The SDGs are ‘Universal, indivisible and interlinked’, and the interdependence between health and development is clearly recognized. Health is seen as both a contributor to, and a beneficiary of, development and is therefore an integral part of all the SDGs. The overriding vision of the SDGs is that ‘all human beings can fulfil their potential in dignity and equality and in a healthy environment’ and ‘enjoy prosperous and fulfilling lives.’^1^

The specific SDG ‘Health Goal’ (Goal No. 3) aims to ‘ensure healthy lives and promote well-being for all at all ages.’ In more detail, target No. 3.3 reads, ‘End the epidemics of AIDS, tuberculosis, malaria and neglected tropical diseases and combat hepatitis, water-borne diseases and other communicable diseases’.^1^ Translated into numerical targets this means that TB incidence and death rates should be reduced by 80% and 90%, respectively, in 15 years. The End TB Strategy has a longer timeline than the SDGs, up to 2035. Although the 80%/90% reduction represents 2030 milestones in the End TB Strategy, the targets are 90% incidence and 95% death rate reduction by 2035, which means that the global burden of TB should be similar to the burden in low-incidence countries today.^2^ This is a substantial shift in ambition level compared to the MDG era (and the related global TB strategy), which aimed solely at reverting the previous upward incidence trend within a 15-year horizon.

Although lumped into one ‘umbrella’ health goal only, the health scope in SDGs has been considerably broadened compared to the MDGs, now encompassing non-communicable diseases, health of the elderly, mental health, substance abuse, tobacco smoking, environmental health hazards, injuries and road traffic accidents.^1^ There has been a true epidemiological transition in the minds behind the new health-related development goals. This is good news for TB care and prevention. The risk of diluting attention to TB by broadening the scope is less than the opportunities to jointly address TB risk factors and common health system bottlenecks.^2–5^ Moreover, the radical shift in ambition level for the TB targets should ensure that there is no room for relaxing the effort.

The WHO's End TB Strategy was developed in parallel with the SDGs, and efforts were made to align indicators and targets. The two stem from the same philosophy: multisectoral approaches are necessary and the root causes of human health despair must be addressed for sustainable impact. Both agendas are bold, responding to an appropriately grand vision and an urgent need for action.

Many of the interventions necessary to reach the ambitious targets set out in the End TB Strategy should now be anchored in the SDGs, especially those that require prominent engagement outside the health sector, such as addressing the social and economic determinants of TB.^4,6^ In short, the End TB Strategy targets cannot be achieved unless rapid and substantial progress is made towards the SDGs. Conversely, better TB care and prevention will help achieve the SDGs in several ways.

A crucial health target is No 3.8: ‘Achieve universal health coverage, including financial risk protection, access to quality essential health-care services and access to safe, effective, quality and affordable essential medicines and vaccines for all’.^1^ TB coverage indicators (TB detection and treatment success) are part of the metrics to monitor progress towards this target, given they are recognized as an essential health intervention for a disease that disproportionately affects the poorest.^7^ The End TB Strategy indicator on ‘catastrophic costs due to TB’ could complement monitoring of the progress towards social protection too.^8^ In the background analysis for the SDGs, TB diagnosis and treatment were listed among the most cost-effective health interventions, and also one that directly contributes to improved productivity and, therefore, overall societal development.^9^ Not by chance, *The Economist* ranked TB interventions as the top health measure and the sixth in the general list of ‘no brainers’ for development investments, based on estimated societal benefit per dollar spent for various development targets.^10^

TB as contributor to development is thus well captured in the SDGs. It is not difficult to justify the fact that TB can also benefit from the SDG achievements. Examples of actions that would address key TB determinants^6^ are clear-cut in the new goals: ending poverty in all its forms everywhere; ending hunger, achieving food security and improved nutrition, and promoting sustainable agriculture; ensuring inclusive and equitable quality education and promoting lifelong learning opportunities for all; achieving gender equality; ensuring access to affordable, reliable, sustainable and modern energy for all; reducing inequality within and among countries; and making cities and human settlements inclusive, safe, resilient and sustainable.

One theme that could have been better emphasised in the SDGs is health research. Goal 9 includes to ‘… foster innovation’ and one of the Goal 3 targets is about ‘strengthening research for medicines and vaccines’. However, investments in broader health research, including epidemiology, social science and health systems research, and the required infrastructure, training and career paths are not clearly outlined. Without it, the path forward will be winding. The third pillar of the End TB strategy addresses this need, and a global action framework has been developed to foster its implementation, promote capacity building in TB endemic countries and stimulate multidisciplinary research along the continuum from basic science to operational research.^11^

Now that the principles and standards have been established, we must ensure that the End TB strategy is fully rolled-out and goals are achieved. The challenges ahead are multiple, but one over-arching need is clear: both the SDGs and End TB Strategy implementation will require very active engagement of all sectors and partners, from the highest level government officials to civil society. This is why WHO makes a point of defining the target audience for the End TB Strategy in much broader terms than for previous global TB strategies. The question at this point is if stakeholders, especially those outside of the health sector, are ready to act rapidly and put the principles into practice. The SDGs promise to strengthen the required institutional, infrastructure and partnership foundations to make goals achievable. Goal 16 is to ‘Promote peaceful and inclusive societies for sustainable development, provide access to justice for all and build effective, accountable and inclusive institutions at all levels’, and Goal 17 is to ‘Strengthen the means of implementation and revitalize the global partnership for sustainable development’. The keywords are there: freedom, peace, security, human rights, rule of law, good governance, and commitment to just and democratic societies. It is up to all of us to contribute to the realization of this vision and sustain it. The ultimate goal of TB elimination (less than one TB case per million population) requires that appropriate interventions are implemented everywhere and the underlying drivers are not only addressed, but sustainably addressed.^12^ History teaches us that epidemics re-emerge when neglect, ignorance and carelessness are let to prevaricate commitment, science and care.
